# Dispensabilities of Carbonic Anhydrase in Proteobacteria

**DOI:** 10.1155/2012/324549

**Published:** 2012-05-15

**Authors:** Kenji Ueda, Hiromi Nishida, Teruhiko Beppu

**Affiliations:** ^1^Life Science Research Center, College of Bioresource Sciences, Nihon University, 1866 Kameino, Fujisawa 252-0880, Japan; ^2^Agricultural Bioinformatics Research Unit, Graduate School of Agriculture and Life Sciences, The University of Tokyo, Bunkyo-ku, Tokyo 113-8657, Japan

## Abstract

Carbonic anhydrase (CA) (E.C. 4.2.1.1) is a ubiquitous enzyme catalysing interconversion between CO_2_ and bicarbonate. The irregular distribution of the phylogenetically distinct classes of CA in procaryotic genome suggests its complex evolutionary history in procaryotes. Genetic evidence regarding the dispensability of CA under high-CO_2_ air in some model organisms indicates that CA-deficient microorganisms can persist in the natural environment by choosing high-CO_2_ niches. In this study, we studied the distribution of CA in the genome of Proteobacteria. While a large majority of the genome-sequenced Proteobacteria retained a CA gene(s), intracellular bacterial genera such as *Buchnera* and *Rickettsia* contained CA-defective strains. Comparison between CA-retaining and CA- deficient genomes showed the absence of whole coding sequence in some strains and the presence of frameshifted coding sequence in other strains. The evidence suggests that CA is inactivated and lost in some proteobacteria during the course of evolution based on its dispensability.

Carbonic anhydrase (CA) (EC 4.2.1.1) is a ubiquitous enzyme catalysing interconversion between CO_2_ and bicarbonate (HCO_3_^−^) [1, 2]. CA is fundamental to various biological functions including photosynthesis, respiration, and CO_2_ transport. To date, the existence of 3 major classes (alpha, beta, and gamma) of this enzyme has been known. Interestingly, no significant structural similarities are observed among these classes. Based on this feature, CA is recognised as an excellent example of convergent evolution [1, 2]. Most of the mammalian and plant CA specifically belong to alpha and beta class, respectively. On the other hand, the distribution of CA in procaryotes is irregular; some retain multiple classes of CA or multiple enzymes from the same class, and others do not retain any class of CA. Hence, it is likely that the evolution of CA function in procaryotes has a complex historical background [1].

Recently, a significant insight into the role of procaryotic CA has been provided by genetic studies in some model organisms such as *Ralstonia eutropha* [3] *Escherichia coli* [4], and *Saccharomyces cerevisiae* [5]. Knockout mutants for CA of these microorganisms are unable to grow under ambient air but normally grow under an atmosphere with high levels (1–5%) of CO_2_.

This phenomenon is explained by the necessity of bicarbonate in the reaction catalysed by several housekeeping enzymes such as phosphoenolpyruvate carboxylase, carbamoyl phosphate synthase, and acetyl-CoA carboxylase [4, 5]. CA-positive microorganisms can generate bicarbonate from environmental CO_2_ by the catalytic reaction of CA and supply it to these enzymes, but CA-negative ones cannot. Hence, the former can grow even under ambient air containing a low level of CO_2_ (0.035%), but the latter cannot initiate growth unless they are supplied with a sufficient concentration of bicarbonate. The latter organisms, however, can grow under a high-CO_2_ atmosphere since it generates a high concentration of bicarbonate to maintain natural equilibrium. This in turn indicates that CA is not essential for microbial growth under high-CO_2_ environments, such as in soil, seawater, intestine, and some other syntrophic and commensal situations. Our previous study showed that an *E. coli* CA mutant was able to grow even under ambient air when it was cocultured with *Bacillus subtilis* [6].

The above-mentioned knowledge makes us speculate that the study of CA distribution in microbial genome will provide an insight into the history of adaptation of microorganisms to environment. Recently, we described that *Symbiobacterium thermophilum*, a unique syntrophic bacterium that effectively grows in coculture with a cognate *Geobacillus stearothermophilus* [7], lost CA in the course of evolution [8]. Our studies have shown that *S. thermophilum* grows on high CO_2_ supply from environment and that this could be the reason for the absence of CA from its genome [6]. The phylogeny of CA distributed in Clostridia to which *S. thermophilum* belongs indicated that the common ancestor of this group of bacteria retained a CA gene and that *S. thermophilum* lost CA in the course of its adaptation to high CO_2_ environments [8].

To deepen our insight into the correlation between CA deficiency and adaptation to high CO_2_ environments, we studied the distribution of CA in the phylum Proteobacteria. Proteobacteria consists of five distinctive classes (alpha, beta, gamma, delta, and epsilon) and unclassified classes including the genus *Magnetococcus* (http://www.ncbi.nlm.nih.gov/genome/). To date (February 1, 2012), complete genome sequence information is available with regard to 649 strains of 249 genera (supplementary Table  S1 in Supplementary Material available online at doi: 10.1155/2012/324549). Our search for the presence of CA by using the pathway database available at GenomeNet (http://www.genome.jp/) and BLAST searches (protein-protein searches based on BLOSUM62 scoring matrix) using known protein sequences annotated to be CA (corresponding to the protein encoded by the intact CA coding sequences shown in Figure 1) as queries showed that 39 strains of 20 genera (Table 1) of the genome-sequenced Proteobacteria do not retain any gene encoding CA.

Among the CA-deficient 20 genera, 4 genera (*Buchnera*, *Blochmannia*, *Rickettsia*, and *Orientia*) contained multiple CA-deficient strains (Table 1). These were obligate intracellular bacteria. It is known that endosymbionts lack genes involved in primary metabolism. For example, *Buchnera* sp. lacks amino acid biosynthesis genes, which are compensated for by the activity of the host organism [9]. Such genetic defects in symbionts genome have probably occurred after establishing a tight, symbiotic relationship with the host organism. Presumably, the intracellular environments contain a high level of CO_2_; hence the catalytic function of CA is not necessary for the bacteria habituating in such environments. On the other hand, some intracellular bacteria such as *Wolbachia* retain a putative CA gene (supplementary Table  S1). This suggests that intracellular environment does not always compensate for CA deficiency.

13 out of the 20 CA-deficient genera contained only a single genome-sequenced strain (supplementary Table  S1). They include intracellular *Candidatus* bacteria and lithoautotrophic and sulfate-reducing bacteria. It is not yet known whether the defect is a common feature of the genus or not, but it is possible that the CA deficiency is widespread among those intracellular bacteria as in the abovementioned genera.

Contrasting to the genuswide deficiency, the other 3 genera (*Actinobacillus*, *Acidithiobacillus*, and *Bartonella*) harboured strain-specific defect of CA (supplementary Table  S1). In *Acidithiobacillus*, all strains except for *Acidithiobacillus caldus* retained the CA gene in the conserved gene cluster (the corresponding region of *Acidithiobacillus ferrooxidans* ATCC 23270 is shown in Figure 1(a)). Contrasting to this, *A. caldus* partially retained the conserved genes. While the genes upstream of CA were conserved, those downstream of CA including CA gene were not (Figure 1(a)). This makes us think of the possibility that the CA deficiency in *A. caldus* is due not to simple deletion but to a genetic rearrangement that has occurred in a relatively large scale.

Lack of CA gene in a conserved gene cluster was also observed with respect to the two species of *Helicobacter*, *Helicobacter felis* and *Helicobacter bizzozeronii*. All the genome-sequenced *Helicobacter* strains except for the two species contained the conserved gene cluster consisting of 6 coding sequences including CA gene (the corresponding region of *Helicobacter pylori* 26695 is shown in Figure 1(a)). Contrasting to this, the genome of the two *Helicobacter* spp. retained the conserved cluster lacking the coding region for CA (the corresponding region of *H. felis* is shown in Figure 1(a)). *H. felis* and *H. bizzozeronii* retained a CA gene in a different locus (corresponding to HFELIS_06160 and HBZC1_14670, resp.).

The other case of strain-specific CA deficiency was based on mutations in the coding sequence. Frameshift mutations inactivating CA gene were identified with respect to the four strains, *Actinobacillus pleuropneumoniae* JL03, *Rickettsia heilongjiangensis*, *Rickettsia japonica,* and *Bartonella quintana* (Figure 1(b)). These organisms retained a frame-shifted coding sequence exactly at the position corresponding to the locus where the intact CA ortholog is located in related strains (Figure 1(b)). The coding region of *A. pleuropneumoniae* JL03 and *R. japonica* contained a single-base deletion in the middle part (supplementary Figures  S1 and S2). The coding region of *R. heilongjiangensis* lacked 95 bp corresponding to the N-terminal part of CA (supplementary Figure  S2). *B. quintana* contained multiple mutations including two single-base deletions, one 8-base insertion, two single-base insertions, and one non-sense (ochre) mutation (supplementary Figure  S3).

It is most likely that the abovementioned mutations inactivating the CA gene have been introduced into the ancestral intact coding sequence during the course of evolution. The diverged mode of mutation may reflect the process of how dispensable genes are lost from the bacterial genome. The existence of the strains carrying the inactivated coding sequence strongly suggests that the CA gene is not necessary for their persistency. It is not yet known how these mutant strains compensate for their CA deficiency, but we may reasonably speculate that it is correlated with the environmental CO_2_ content.

The CA-deficient genera described in this paper are usually handled under a microaerobic or anaerobic atmosphere containing 1–5% CO_2_ [10]. Hence, the conventional isolation method for these organisms has made possible isolation of strains requiring high CO_2_. On the other hand, the standard isolation procedure for aerobic proteobacteria using ambient air prevents isolation of CO_2_-requiring strains. This makes us think of the possibility that the very high proportion of CA-positive strains (610 out of 649 strains) (supplementary Table  S1) is due to the limitation of isolation condition and is not appropriately reflecting the true distribution of CA in Proteobacteria.

The evolution of microbial genome reflects the history of environmental change. We expect that comprehensive analyses regarding the distribution of specific adaptive functions in microbial genome will provide deep insights into the constitution of the ecosystem.

## Supplementary Material

Supplementary figure S1. Frameshift mutation in *A. plueropneumoniae* JL03 strain. Supplementary figure S2. Frameshift mutation in *R. heilongjiangensis* and *R. japonica*. Supplementary figure S3. Frameshift mutations in *B. quintana*.
Supplementary table S1. The list of genome-sequenced proteobacteria. The organisms that do not retain any carbonic anhydrase gene are marked in yellow. The list is based on the data available at GenomeNet web site http://www.genome.jp/.

## Figures and Tables

**Figure 1:**
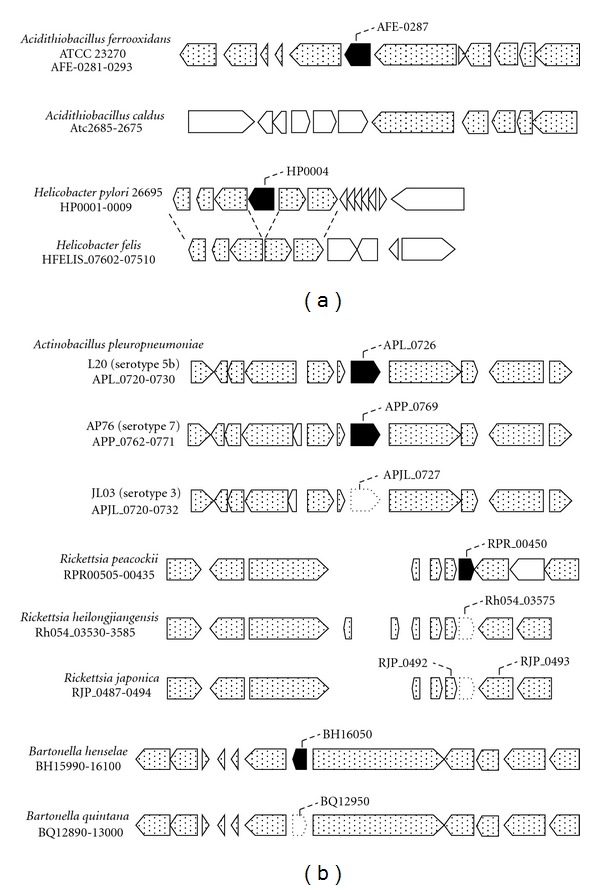
Comparison of the conserved gene cluster containing CA gene between CA-retaining and CA-deficient strains affiliating with the same genus of proteobacteria. Strains lacking whole CA coding sequences (a) and retaining frameshifted CA coding sequences (b) are compared with those retaining intact CA genes of the same genus. The coding sequence for CA and other conserved genes are shown in solid and dotted bars, respectively.

**Table 1:** CA-deficient strains of genome-sequenced Proteobacteria.

Class	Genus*	Species/strain
Gammaproteobacteria	*Buchnera* (7)	*Buchnera aphidicola* APS
		*Buchnera aphidicola* Sg
		*Buchnera aphidicola* Bp
		*Buchnera aphidicola *Cc
		*Buchnera aphidicola* 5A
		*Buchnera aphidicola* Tuc7
		*Buchnera aphidicola* (*Cinara tujafilina*)
	*Wigglesworthia* (1)	*Wigglesworthia glossinidia*
	*Blochmannia* (3)	*Candidatus Blochmannia floridanus*
		*Candidatus Blochmannia pennsylvanicus*
		*Candidatus Blochmannia vafer*
	*Riesia* (1)	*Candidatus Riesia pediculicola*
	*Moranella* (1)	*Candidatus Moranella endobia*
	*Actinobacillus* (4)	*Actinobacillus pleuropneumoniae* JL03 (serotype 3)
	*Thioalkalimicrobium* (1)	*Thioalkalimicrobium cyclicum*
	*Acidithiobacillus* (1)	*Acidithiobacillus caldus*
	*Baumannia* (1)	*Baumannia cicadellinicola*
	*Carsonella* (1)	*Candidatus Carsonella ruddii*

*Betaproteobacteria*	*Zinderia* (1)	*Candidatus Zinderia insecticola CARI*

Deltaproteobacteria	*Desulfohalobium* (1)	*Desulfohalobium retbaense*
	*Desulfococcus* (1)	*Candidatus Desulfococcus oleovorans*
	*Desulfatibacillum* (1)	*Desulfatibacillum alkenivorans*
	*Syntrophobacter* (1)	*Syntrophobacter fumaroxidans*
	*Hippea* (1)	*Hippea maritima*

Alphaproteobacteria	*Rickettsia* (15)	*Rickettsia prowazekii*
		*Rickettsia typhi*
		*Rickettsia canadensis*
		*Rickettsia conorii*
		*Rickettsia akari*
		*Rickettsia rickettsii Sheila Smith*
		*Rickettsia rickettsii Iowa*
		*Rickettsia massiliae*
		*Rickettsia heilongjiangensis*
		*Rickettsia japonica*
		*Rickettsia bellii *RML369-C
	*Orientia* (2)	*Orientia tsutsugamushi *Boryong
		*Orientia tsutsugamushi* Ikeda
	*Bartonella* (6)	*Bartonella quintana*
	*Hodgkinia* (1)	Candidatus *Hodgkinia cicadicola *

*The number of genome-sequenced species/strains of each genus is shown in parentheses.
