# Supplementary material for: Dispensabilities of Carbonic Anhydrase in Proteobacteria
Source: Int J Evol Biol. 2012 May 15;2012:324549. doi: 10.1155/2012/324549 (PMC3364446; doi:10.1155/2012/324549)
Supplement: Supplementary file 1 — Supplementary figure S1. Frameshift mutation in A. plueropneumoniae JL03 strain. Supplementary figure S2. Frameshift mutation in R. heilongjiangensis and R. japonica. Supplementary figure S3. Frameshift mutations in B. quintana. Supplementary table S1. The list of genome-sequenced proteobacteria. The organisms that do not retain any carbonic anhydrase gene are marked in yellow. The list is based on the data available at GenomeNet web site http://www.genome.jp/. [file 324549.f1.doc]

**Supplementary figure S1.** Frameshift mutation in *A. plueropneumoniae* JL03 strain. The intact CA-coding sequence of *A. plueropneumoniae* L20 (RPK_0726; APL) (1-717 nt; the start and stop codons are highlighed) was used as a query for the BLASTN search in *A. plueropneumoniae* JL03 (APJL) genome database. The site of possible frameshift mutation is indicated by an arrow.

^f^Met

APL: 1 atgaaacagatagaaaaattgtttgccaataaccatgcttgggcaactcggatgaaagac 60

||||||||||||||||||||||||||||||||||||||||||||||||||||||||||||

APJL: 798568 atgaaacagatagaaaaattgtttgccaataaccatgcttgggcaactcggatgaaagac 798627

APL: 61 gaacagtcggattattttaaacaacttgccgagcatcaaaagccgacttatctttggatt 120

||||||||||||||||||||||||||||||||||||||||||||||||||||||||||||

APJL: 798628 gaacagtcggattattttaaacaacttgccgagcatcaaaagccgacttatctttggatt 798687

APL: 121 ggctgttcggacagtcgtgttccggccgaaaaattaaccggtttaggtccgggcgagctt 180

||||||||||||||||||||||||||||||||||||||||||||| ||||||||||||||

APJL: 798688 ggctgttcggacagtcgtgttccggccgaaaaattaaccggtttaagtccgggcgagctt 798747

APL: 181 tttgttcatcgtaacgtggcaaatttagttatccataccgatctgaattgtctttccgtc 240

||||||||||||||||||||||||||||||||||||||||||||||||||||||||||||

APJL: 798748 tttgttcatcgtaacgtggcaaatttagttatccataccgatctgaattgtctttccgtc 798807

APL: 241 gtgcaatatgcggtagatgtattggagatcgaacatattattatttgcggtcataccaac 300

||||||||||||||||||||||||||||||||||||||||||||||||||||||||||||

APJL: 798808 gtgcaatatgcggtagatgtattggagatcgaacatattattatttgcggtcataccaac 798867

APL: 301 tgcggtggtatccaagctgcaattggtactgtcgaagattacggtttaatcagcaactgg 360

||||||||||||||||||||||||||||||||||| ||||||||||||||||||||||||

APJL: 798868 tgcggtggtatccaagctgcaattggtactgtcga-gattacggtttaatcagcaactgg 798926

-1 bp

APL: 361 ttattgcatattcgtgatctgtggtttaaacacagctatttactcggtaatcttccctcc 420

||||||||||||||||||||| ||||||||||||||||||||||||||||||||||||||

APJL: 798927 ttattgcatattcgtgatctgcggtttaaacacagctatttactcggtaatcttccctcc 798986

APL: 421 gagcaacgtgccaatatgcttacccgcttaaacgttgccgagcaagtttataacttaggg 480

||||||||||||||||||||||||||||||||||||||||||||||||||||||||||||

APJL: 798987 gagcaacgtgccaatatgcttacccgcttaaacgttgccgagcaagtttataacttaggg 799046

APL: 481 cgcagttctattgttaccgccgcttggaagcgaggtaagaaactctcgattcacggctgg 540

||||||||||||||||||||||||||||||||||||||||||||||||||||||||||||

APJL: 799047 cgcagttctattgttaccgccgcttggaagcgaggtaagaaactctcgattcacggctgg 799106

APL: 541 gtatatgatgttaatgacggtttcttaattgatcagggggtaatcgcaaccagtacggaa 600

||||||||||||||||||||||||||||||||||||||||||||||||||||||||||||

APJL: 799107 gtatatgatgttaatgacggtttcttaattgatcagggggtaatcgcaaccagtacggaa 799166

APL: 601 acactcgaaattacttatcgaaatgcgattgcgaaacttgcgacggaagtggaagaaatg 660

||||||||||||||||||||||||||||||||||||||||||||||||||||||||||||

APJL: 799167 acactcgaaattacttatcgaaatgcgattgcgaaacttgcgacggaagtggaagaaatg 799226

stop

APL: 661 atcgccaataaacccgtcgaacaatcaaatccaattgaaaataatcatattgattaa 717

|||||||||||||||||||||||||||||||||||||||||||||||||||||||||

APJL: 799227 atcgccaataaacccgtcgaacaatcaaatccaattgaaaataatcatattgattaa 799283

**Supplementary figure S2.** Frameshift mutation in *R. heilongjiangensis* and *R. japonica*. The intact CA-coding sequence of *R. peacockii* (RPR_00450; RPK) (1-516 nt; the start and stop codons are highlighed) was used as a query for the BLASTN search in *R. heilongjiangensis* (RHE) and *R. japonica* (RJA) genome database. The site of possible frameshift mutation is indicated by an arrow.

^f^Met

RPK: 1 atgctcattatcccttacaaaggagttacgccaagaatcgataaaa 46

||||||||||||||||||||||| ||||| ||||||||||||||||

RHE: 627411 atgctcattatcccttacaaaggggttacaccaagaatcgataaaa 627456

-95 bp

RPK: 142 ataggaaataatactaacgtacaagacggtagtgtaattcatgcttca 201

||||||||||||||||||||||||||||||||||||||| || |||||

RHE: 627457 ataggaaataatactaacgtacaagacggtagtgtaatttatacttca 627504

RPK: 202 aggtttaacggaccggtagaaataggagataatataactatcggtcatctctctcttatt 261

|| ||||| |||||||||||||||| || |||||||||||||||||||||||||

RHE: 627505 agatttaatagaccggtagaaataggcga------aactatcggtcatctctctcttatt 627558

RPK: 262 catgcctgtacaatacataataatgcttttatcggtatgagtgctacgataatggattat 321

||||||||||||||||||||||||||||||||||||||||||||||||||||||||||||

RHE: 627559 catgcctgtacaatacataataatgcttttatcggtatgagtgctacgataatggattat 627618

RPK: 322 gcagtaatagaagaatatgcttttattgctgcaagcagtcttatcctgccaaagaagata 381

||||||||||||||| ||||||||||||||||| |||||||||||| |||||||||||||

RHE: 627619 gcagtaatagaagaacatgcttttattgctgcaggcagtcttatcccgccaaagaagata 627678

RPK: 382 attaaatctcaagaattatggatgggatcccctgcaaaatttgttagatatttaaccgat 441

||||||||||||||||||||||||||||||||||||||||||||||||||||||||||||

RHE: 627679 attaaatctcaagaattatggatgggatcccctgcaaaatttgttagatatttaaccgat 627738

RPK: 442 caagatttagaatatatgcaagataatgtaagacattatgtagaacttgcaaatgtttat 501

||||||||||||||||||||||||||||||||| ||||||||||||||||||||||||||

RHE: 627739 caagatttagaatatatgcaagataatgtaagaaattatgtagaacttgcaaatgtttat 627798

stop

RPK: 502 aagatactcgtttaa 516

|||||||||||||||

RHE: 627799 aagatactcgtttaa 627813

**Supplementary figure S2 (continued).**

^f^Met

RPK: 1 atgctcattatcccttacaaaggagttacgccaagaatcgataaaagtgcatatattgcc 60

||||||||||||||||||||||| ||||| |||||||||||||||| |||||||||| ||

RJA: 628951 atgctcattatcccttacaaaggggttacaccaagaatcgataaaaatgcatatattacc 629010

RPK: 61 gaaagcagctctttaataggagatgttgaaataggtagtaattcaagcatttggtttaat 120

|||||||||||||||||||||||||||||||||||||||||||||||||||||||||||

RJA: 629011 aaaagcagctctttaataggagatgttgaaataggtagtaattcaagcatttggtttaat 629070

RPK: 121 acggttcttagaggcgacgttgaatcgataaaaataggaaataatactaacgtacaagac 180

|||||||||||||||||||||||||||||||||||||||||||||||||||||||||||

RJA: 629071 acggttcttagaggcgacgttgaatcgataaaaataggaaataatactaacgtacaagat 629130

RPK: 181 ggtagtgtaattcatgcttcaaggtttaacggaccggtagaaataggagataatataact 240

|||||||||||| || ||||||| ||||| ||| |||||||||||| || 　||||

RJA: 629131 ggtagtgtaatttatacttcaagatttaatagac-ggtagaaataggcga------aact 629183

　　　　　　　　　　　　　　　　　　　　　　　　　　　　　 -1 bp

RPK: 241 atcggtcatctctctcttattcatgcctgtacaatacataataatgcttttatcggtatg 300

||||||||||||||||||||||||| ||||||||||||||||||||||||||||||||||

RJA: 629184 atcggtcatctctctcttattcatgtctgtacaatacataataatgcttttatcggtatg 629243

RPK: 301 agtgctacgataatggattatgcagtaatagaagaatatgcttttattgctgcaagcagt 360

|||||||||||||||||||||||||||||||||||| ||||||||||||||||| |||||

RJA: 629244 agtgctacgataatggattatgcagtaatagaagaacatgcttttattgctgcaggcagt 629303

RPK: 361 cttatcctgccaaagaagataattaaatctcaagaattatggatgggatcccctgcaaaa 420

||||||| ||||||||||||||||||||||||||||||||||||||||||||||||||||

RJA: 629304 cttatcccgccaaagaagataattaaatctcaagaattatggatgggatcccctgcaaaa 629363

RPK: 421 tttgttagatatttaaccgatcaagatttagaatatatgcaagataatgtaagacattat 480

||||||||||||||||||||||||||||||||||||||||||||| |||||||| |||||

RJA: 629364 tttgttagatatttaaccgatcaagatttagaatatatgcaagatcatgtaagaaattat 629423

stop

RPK: 481 gtagaacttgcaaatgtttataagatactcgtttaa 516

||||||||||||||||||||||||||||||||||||

RJA: 629424 gtagaacttgcaaatgtttataagatactcgtttaa 629459

**Supplementary figure S3.** Frameshift mutations in *B. quintana*. The intact CA-coding sequence of *B. henselae* (BH16050; BHE) (1-657 nt; the start and stop codons are highlighed) was used as a query for the BLASTN search in *B. quintana* (BQU) genome database. The sites of possible frameshift mutation and non-sense mutation are indicated by arrows.

^f^Met

BHE: 1 atgacgcgtttaccagaaagactcttaagcggttaccggtcttttataaaaaatcatttt 60

||||||||||||||||||| ||| |||||| ||| | ||| |||| |||||||||||||

BQU: 1505859 atgacgcgtttaccagaaaaacttttaagcatttatcagtcgtttacaaaaaatcatttt 1505800

BHE: 61 ttttataaaacggcacattatcagcaattagcaatagaggggcagaagcctgaaattttg 120

| | ||||||| || ||| ||||||||| || || || | |||||| ||||| ||||

BQU: 1505799 tcatataaaacagcctattgtcagcaattggcgattgaagagcagaaatctgaagtttta 1505740

BHE: 121 gtcattgcttgttgtgattcgcgggcaataccagaaacgatttttgatgctaaaccaggt 180

||||||||||||||| ||||||||| | | ||| ||||||||||| ||||||||| ||||

BQU: 1505739 gtcattgcttgttgtaattcgcgggtagtgccaaaaacgattttttatgctaaactaggt 1505680

BHE: 181 gaaatttttacgc-tgcgcaacgtggcaaatttggttcctcctttttc-tcctgataatc 238

||||||||||||| ||||||||||||||||||| ||||||| ||||| || |||||| |

BQU: 1505679 gaaatttttacgcctgcgcaacgtggcaaatttagttcctctttttttgtcttgataacc 1505620

+1 bp +1 bp

BHE: 239 agtatcatgcaacatcagcagcgattgaatatgctgtccaattgcttgaggtgaagcata 298

| ||||| |||||||||||||||||| || |||| || ||||| |||||||| |||||||

BQU: 1505619 aatatcacgcaacatcagcagcgattaaacatgcggtgcaattacttgaggtcaagcata 1505560

BHE: 299 ttgttgtttttggtcatgcccattgtggggggg--------tgaacactgctcttgaggg 350

|||||||||||||| ||| ||||||||||| || ||| ||||| |||||| ||

BQU: 1505559 ttgttgtttttggttatggccattgtggggaggggctgagctgagcactgttcttgaagg 1505500

+8 bp

BHE: 351 gacgtgcaagtctttatcgtcaaatgattttattggtcaatggataagtcttttgatacc 410

|||| |||| ||||||||||||||||||||||||||||||||||||||| ||||| |||

BQU: 1505499 gacgggcaaatctttatcgtcaaatgattttattggtcaatggataagt-ttttggcacc 1505441

-1 bp

BHE: 411 agcagcacaaacaattattgaaaacaagtcgttgaccccttcagagcagcagaccgcatt 470

||||||||||||| || ||| || || || ||||| |||||||||||||||||||

BQU: 1505440 agcagcacaaacagttgttggcaataaatcattgactgggccagagcagcagaccgcatt 1505381

BHE: 471 agagaaactttctattcgtcattcgttgaaaaatttagaaacatttccctggataaaggc 530

|||| |||||||||||||||||||| ||||||||||||| ||||||||||| ||||||||

BQU: 1505380 agagtaactttctattcgtcattcgctgaaaaatttagagacatttccctgtataaaggc 1505321

Non-sense(orchre)

BHE: 531 gcgtaaagatcagggtattttgacgttgcacggggtttggtttgatatctcaagtggtga 590

| | |||||||| | | |||||||||| ||||||||||||||||||||||||| ||||||

BQU: 1505320 gtgcaaagatcaagatgttttgacgttacacggggtttggtttgatatctcaactggtga 1505261

BHE: 591 attatggagtatggaacaggaaacaggtcattttatgcgtgttgaggtggggaatttt 648

||| |||||| | |||||||||||||| | |||||||||||| || || |||||||||

BQU: 1505260 attgtggagtgtagaacaggaaacaggccgttttatgcgtgtcgatgtcgggaatttt 1505203

stop

BHE: 649 gaggaataa 657

||| |||

BQU: 1505204 -aggcgtaa 1505212

-1 bp
